# The Impact of Age on In-Hospital Mortality in Patients with Sepsis: Findings from a Nationwide Study

**DOI:** 10.3390/jcm14217637

**Published:** 2025-10-28

**Authors:** Ohad Gabay, Ruth Smadar-Shneyour, Shiloh Adi, Matthew Boyko, Yair Binyamin, Victor Novack, Amit Frenkel

**Affiliations:** 1 General Intensive Care Unit, Soroka University Medical Center, Beer-Sheva 84101, Israel; 2 Faculty of Health Sciences, Ben-Gurion University of the Negev, Beer-Sheva 84101, Israel; ruthsm@post.bgu.ac.il (R.S.-S.);; 3 Clinical Research Center, Soroka University Medical Center, Beer-Sheva 84101, Israel; 4 Department of Anesthesiology, Soroka University Medical Center, Beer-Sheva 84101, Israel; 5 Anesthesia, Critical Care and Pain Medicine, Beth Israel Deaconess Medical Center, Harvard Medical School, Boston, MA 02115, USA

**Keywords:** sepsis, in-hospital mortality, age, internal medicine wards, comorbidities, nationwide cohort, spline regression

## Abstract

**Background**: Age is a well-established determinant of sepsis outcomes, often integrated into severity scoring systems. However, most studies focus on critically ill patients in intensive care units (ICUs), with limited insight into how age influences mortality in non-ICU settings, particularly across the full adult lifespan. **Objective**: To investigate the relationship between age and in-hospital mortality in patients with sepsis hospitalized in internal medicine wards, using age-stratified logistic and spline regression models. **Methods**: We conducted a retrospective, multicenter cohort study involving 4300 adult patients admitted to internal medicine wards at eight academic hospitals affiliated with Clalit Health Services in Israel between December 2001 and October 2020. All patients were diagnosed with sepsis during hospitalization and died during their hospital stay. Patients were stratified into seven age groups (18–34, 35–44, 45–54, 55–64, 65–74, 75–84, >85 years). Logistic regression identified age-specific comorbidities associated with mortality. Adjusted spline regression models were used to estimate mortality probabilities across age ranges. **Results**: The cohort had a mean age at death of 78.84 years, and 51.7% were female. Mortality probability increased with age but demonstrated non-linear trends. Sharp fluctuations in predicted mortality were observed in middle-aged groups (especially ages 45–54), with peaks not captured in conventional binary or linear models. Hematologic and solid neoplasms were strongly associated with mortality in younger groups, while cardiovascular comorbidities such as heart failure and atrial fibrillation were more prominent in older adults. **Conclusions**: Age is a major determinant of in-hospital mortality in septic patients on internal medicine wards, but its effect is non-linear and age-specific. Our findings highlight a unique population of patients with severe sepsis not managed in critical care settings and underscore the need for more nuanced, age-stratified risk assessment models outside of the ICU.

## 1. Introduction

Sepsis remains a major cause of morbidity and mortality worldwide, particularly among hospitalized adults. Numerous factors are known to influence outcomes in sepsis, reflecting both patient-related vulnerabilities and the severity of the disease process [1].

One of the most decisive drivers of sepsis prognosis is the severity of the acute illness. The presence of septic shock, multi-organ dysfunction, and the need for vasopressors or invasive mechanical ventilation are powerful indicators of poor outcomes [2,3]. Not only is the need for invasive mechanical ventilation a marker of critical illness, but also the duration of ventilation and the timing of its initiation has been shown to be independently associated with in-hospital mortality in septic patients [4,5].

Laboratory parameters reflecting physiological derangement, including elevated serum lactate, hypoalbuminemia, thrombocytopenia, and metabolic acidosis, have also been associated with increased mortality [1,6,7,8]. Treatment-related factors are also critically important. Timely recognition of sepsis, adherence to early-resuscitation bundles, prompt initiation of appropriate antimicrobial therapy, and effective source control have all been shown to reduce mortality [9,10].

Amid these complex and interacting risk factors, age is frequently cited as a key determinant of prognosis in patients with sepsis. Accordingly, major scoring systems such as APACHE II, SAPS II, and the Charlson Comorbidity Index (CCI) incorporate age as a key component in their prognostic models [11,12,13]. Numerous studies over the past two decades have demonstrated a clear association between increasing age and higher in-hospital mortality, A narrative review reports that in-hospital mortality in patients ≥ 80 years ranges from 40–80%, compared to 30–60% in those ≥65; in the same cohort, mortality among the very elderly was nearly double that seen in patients under 50 (49.3% vs. 25.2%) [14,15,16]. Much of this evidence, however, is derived from critically ill populations in intensive care units (ICUs), where the very high acuity of illness, presence of multi-organ dysfunction, and advanced life-support interventions may amplify the influence of age on outcomes. In these settings, mortality rates often exceed 40% for patients over the age of 80, and studies consistently report higher adjusted odds of death in older age groups [14].

In contrast, patients hospitalized with sepsis in general medical wards, who often present with less dramatic hemodynamic compromise and do not require ICU-level organ support, are underrepresented in the literature. While the burden of sepsis in non-ICU settings is substantial—especially among elderly patients—data on how age affects mortality in this population remain limited. Recent studies have begun to address this gap, highlighting meaningful differences in outcomes between ICU and non-ICU sepsis populations. For instance, Oami et al. (2023) analyzed a large Japanese nationwide database and demonstrated substantial mortality among both ICU and non-ICU sepsis patients, underscoring the need for focused investigation in general wards [17]. However, despite such contributions, the literature remains sparse, and findings are inconsistent regarding whether age remains an independent predictor of mortality after adjusting for illness severity and comorbidity outside ICU environments.

Moreover, most prior studies have assessed age in broad categories—commonly treating age as a binary (e.g., ≥65 vs. <65) or linear predictor—without exploring the full age continuum across adulthood [14,15,16,18,19]. As a result, literature provides little insight into how mortality risk evolves across the younger and middle-aged adult populations, not only among the elderly. Given both the disproportionate focus on ICU cohorts and the lack of detailed age stratification in existing literature, there remains a significant knowledge gap regarding the prognostic role of age in hospitalized septic patients managed on medical wards.

## 2. Objective

This study aimed to evaluate the relationship between age and in-hospital mortality among patients with sepsis admitted to internal medicine wards. To address existing gaps in the literature, we analyzed mortality across seven discrete age categories and applied restricted cubic spline modeling to reveal potential non-linear associations between age and in-hospital mortality risk outside the ICU.

## 3. Methods

### 3.1. Study Population

We conducted a nationwide, multicenter, population-based retrospective cohort study involving patients hospitalized in internal medicine wards at eight academic medical centers affiliated with Clalit Health Services (CHS), the largest healthcare provider in Israel, serving over 4.5 million people.

The study included adult patients admitted between December 2001 and October 2020 with infectious diseases who were diagnosed with sepsis during hospitalization, as defined below. An initial analysis included all patients with sepsis (regardless of outcome) to determine overall in-hospital mortality rates and trends. Subsequently, a subset analysis restricted to patients who died during hospitalization was conducted to characterize factors associated with in-hospital mortality.

Sepsis was defined as the presence of one or more of the following ICD-9 codes in the admission diagnosis or at any point during hospitalization: 038.0, 038.1, 038.2, 038.3, 038.4, 038.8, 038.9, 003.1, 020.2, 022.3, 036.2, 036.3, 054.5, 098.89, 112.5, 995.91, 995.92, and 785.52, as used in previous studies [3].

Specific ICD-9 codes used to define sepsis are listed in Supplementary Table S1.

The study population was stratified into the following age groups: 18–34 years, 35–44, 45–54, 55–64, 65–74, 75–84 and 85+. These specific age groups were selected to be as narrow as possible to accurately discern the variations in age effects within each group.

### 3.2. Data Sources and Clinical Definitions

All data were extracted from patients’ electronic medical records. This included demographic information (age, gender, socioeconomic status), hospitalization details. Data on underlying chronic medical conditions were obtained from CHS disease registries, based on International Classification of Diseases, Ninth Revision, Clinical Modification (ICD-9-CM) codes Supplementary Table S1.

Data were extracted using the CHS data-sharing platform powered by MDClone. The study was conducted in accordance with the Declaration of Helsinki and was approved by the Institutional Review Board of Soroka Medical Center (protocol number 0108-16-SOR). Informed consent was waived due to the retrospective nature of the study. Portions of the manuscript text were rephrased using ChatGPT-5 (OpenAI, 2025) to improve clarity and language fluency.

### 3.3. Statistical Analysis

Descriptive statistics were used to summarize the characteristics of the study population. Categorical variables are presented as frequencies and percentages, and continuous variables are presented as means and standard deviations or medians and interquartile ranges, as appropriate.

To identify potential confounders, unadjusted logistic regression was initially performed within each age group to assess the association between age (as a continuous variable) and in-hospital mortality. Variables significantly associated with in-hospital mortality in these age-specific logistic regression models were considered potential confounders. Subsequently, adjusted spline regression models were used to examine the relationship between age and in-hospital mortality within each age group, controlling for comorbidities significant within each group.

To explore potential non-linear relationships between age and in-hospital mortality, we used adjusted restricted cubic spline (RCS) regression. The spline was modeled with seven knots, placed at the predefined age group boundaries (18–34, 35–44, 45–54, 55–64, 65–74, 75–84, ≥85 years). This specification allowed flexible modeling of age-related risk while maintaining interpretability. The model fit was compared to linear and polynomial regressions using the Akaike Information Criterion (AIC), with the RCS model providing a superior fit. Supplementary Figure S1.

Analyses were performed in R version 4.2.1 using the rms package (function rcs). A two-sided *p* value < 0.05 was considered statistically significant.

#### Outcome

The primary outcome was in-hospital mortality, defined as death occurring during the index hospitalization.

An initial analysis included all patients with sepsis to determine overall in-hospital mortality rates and trends. Subsequently, a subset analysis restricted to patients who died during hospitalization was conducted to identify comorbidities and demographic factors associated with mortality within each age group.

## 4. Results

### 4.1. Patient Characteristics

**Figure 1:**
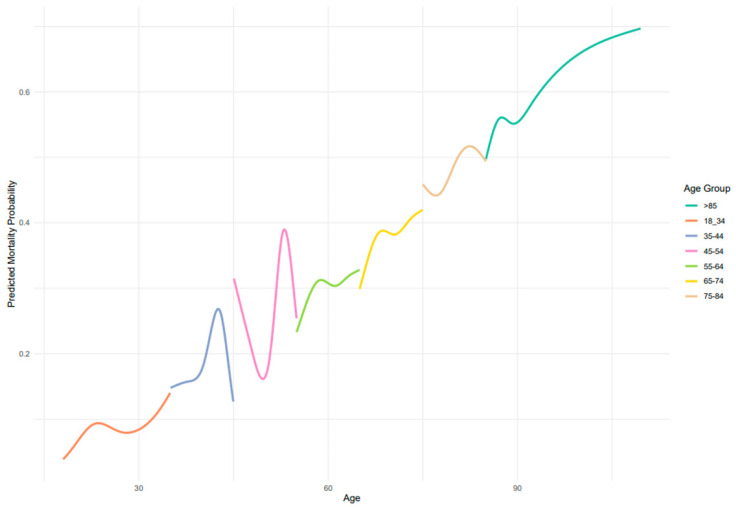
Adjusted spline regression–death during hospitalization (*n* = 4300).

A total of 9805 patients were included in the study, all with a documented diagnosis of sepsis during hospitalization. The initial analysis encompassed all patients with sepsis to assess age-related trends in in-hospital mortality, as shown in Figure 1.

For the subsequent subset analysis, we included only patients who died during hospitalization to characterize mortality predictors within each age group. Baseline characteristics of the study population, stratified by age group, are presented in Table 1**.** The largest age group was 75–84 years (*n* = 1516), while the smallest was 18–34 years (*n* = 30). Slightly more than half of the cohort were female (51.7%). The mean age at death was 78.84 years (SD, 12.35), and the average length of hospital stay was 10.78 days (SD, 7.12).

### 4.2. Logistic Regression Analysis

Unadjusted logistic regression analyses were performed within each age group among patients who died during hospitalization to identify comorbidities associated with mortality. The results are summarized in Table 2.

Across most age groups, malignancy (solid or hematologic) and chronic organ dysfunction (particularly cirrhosis, COPD, CKD, and CHF) were consistently associated with higher odds of in-hospital death.

In younger adults (18–54 years), neoplastic diseases and cirrhosis showed the strongest associations, while in middle-aged groups (55–74 years), cerebrovascular disease and chronic organ failure (renal, hepatic, or pulmonary) emerged as prominent predictors.

Among the oldest patients (≥75 years), cardiovascular comorbidities such as congestive heart failure and atrial fibrillation became more influential.

Hypertension showed an inverse association with mortality in the 65–74-year group, and male sex was associated with lower mortality odds among those aged ≥ 85 years.

These patterns indicate that while the specific predictors vary by age, chronic disease burden—particularly malignancy and organ dysfunction—remains a major determinant of in-hospital death across all strata.

### 4.3. Spline Regression Analysis

Figure 1 displays the predicted probability of in-hospital mortality across age groups, as estimated by adjusted spline regression models. Overall, there was a trend of increasing mortality probability with advancing age, although the relationship was non-linear and varied across specific age ranges Mortality remained lowest among patients aged 18–34 years and rose progressively from midlife onward, reaching its highest levels in patients over 85 years. Intermediate age groups (45–74 years) demonstrated modest fluctuations, consistent with the patterns shown in Figure 1.

## 5. Discussion

This nationwide, multicenter study provides a detailed age-stratified evaluation of in-hospital mortality among patients with sepsis hospitalized in internal medicine wards, extending current knowledge beyond the ICU-focused literature. We observed a non-linear association between age and mortality, with particularly high rates among patients aged ≥85 years (predicted mortality exceeding 60%) [14,15,16]. These findings highlight that the steep age-related increase in sepsis mortality, which is well documented in ICU populations [7], also persists in non-ICU settings—where patients receive limited organ support yet experience a substantial mortality burden.

Crucially, our age-stratified spline regression models demonstrated that mortality risk does not increase uniformly across age groups. Among younger adults (18–44 years), predicted mortality remained relatively low, though a distinct peak was observed around the age of 40.

In middle-aged adults (45–54 years), mortality probabilities fluctuated with age, revealing sharp peaks and dips that are not captured when age is treated as a linear or binary variable—as is common in many prognostic models and epidemiological studies [14,15,17,18,19]. This reinforces the importance of avoiding oversimplified age categories when examining sepsis outcomes. Several possible medical explanations may underlie these non-linear patterns. First, this age group may reflect a heterogeneous population in terms of baseline health, with some individuals beginning to accumulate significant comorbidities—such as cirrhosis, chronic kidney disease, or hematologic malignancies—that substantially increase vulnerability to sepsis-related mortality. Others in this group may still be relatively healthy, leading to divergent outcomes. Additionally, delays in diagnosis or care-seeking behavior may be more common in middle-aged adults, who may not perceive themselves to be at high risk, thereby contributing to poorer outcomes in a subset of patients. Furthermore, health system factors such as lower prioritization for ICU admission compared to younger or older individuals may play a role in mediating access to advanced care. The observed midlife mortality peak should be interpreted with caution given the relatively smaller sample size within certain age strata and the retrospective nature of administrative data. The observed fluctuations may therefore reflect the interplay between emerging chronic illness, behavioral factors, and variations in healthcare delivery specific to this transitional life stage.

It is important to emphasize that the population described in this study represents a unique and underrecognized group: patients with sepsis who were not admitted to an intensive care unit yet ultimately died during hospitalization. This raises critical questions about disease severity, treatment decisions, and triage processes. Particularly in younger and middle-aged adults, one might expect that patients with sepsis severe enough to result in death would typically be transferred to critical care settings for more aggressive management. The fact that these patients remained on general medical wards suggests either atypical clinical presentations, potential delays in deterioration recognition, or decisions against escalation-possibly due to perceived futility or comorbidity burden. These contextual factors should be carefully considered when interpreting our findings, especially regarding younger age groups in whom ICU admission would typically be anticipated for life-threatening infections.

Another key finding of this study is that different comorbidities play varying roles in predicting mortality across age groups. For example, hematologic and solid neoplasms were highly predictive of mortality in the youngest cohorts, while cardiovascular diseases (e.g., congestive heart failure and atrial fibrillation) were more prominent among older adults. These findings suggest that age interacts with other risk factors in shaping the clinical trajectory of sepsis, emphasizing the need for age-adapted risk assessments and management strategies.

Our results also highlight the relative lack of prior research examining the full adult age continuum in sepsis outcomes. While most previous studies focused on ICU patients and often dichotomized age (e.g., ≥65 vs. <65) [14,15,17,18], our approach using seven discrete age groups uncovered nuanced differences that may inform future research and clinical practice. Importantly, the middle-aged population (ages 45–64), often overlooked in sepsis mortality literature, demonstrated heterogeneous mortality patterns and associations with distinct comorbidities such as cirrhosis and chronic kidney disease.

This study’s strengths include its large, nationwide sample and focus on internal medicine wards—a patient population often underrepresented in sepsis research. Importantly, the observed mortality patterns may reflect a combination of comorbidity burden, delayed recognition, and resource limitations that disproportionately affect non-ICU patients. These findings highlight the critical importance of early identification and timely escalation of care in general medical wards, where patients may have substantial physiological reserve but receive less intensive monitoring.

Several limitations should be acknowledged. The retrospective design restricts causal inference. Although multiple comorbidities were adjusted for, residual confounding cannot be excluded, particularly given the absence of standardized severity-of-illness scores (e.g., SOFA, APACHE II). In addition, detailed treatment-related data (such as antibiotic timing, fluid resuscitation, vasopressor use, mechanical ventilation, or source control procedures) were not available, limiting our ability to account for therapeutic variability across age groups.

Because multiple statistical comparisons were performed, we did not apply formal corrections (e.g., Bonferroni) to avoid excessive type II error and the loss of potentially important signals in this exploratory analysis. As a result, some associations may represent chance findings, and the results should therefore be interpreted with appropriate caution. The small number of cases in the youngest age strata may have reduced statistical precision.

Finally, the use of ICD-9 codes to identify sepsis cases may have led to underestimation or misclassification of some patients.

In conclusion, this study demonstrates that age is a major determinant of in-hospital mortality among septic patients in general medical wards, but the risk does not increase in a simple linear fashion. Age-specific mortality patterns and comorbidity profiles vary substantially across the adult lifespan. These findings call for more nuanced age-stratified analyses in future sepsis research and underscore the importance of developing tailored clinical pathways for older adults, who bear a disproportionate share of the sepsis burden even outside of the ICU.

## Figures and Tables

**Table 1:** Study group baseline characteristics.

Characteristics	Overall
N	4300
Age (SD)	78.81 (12.35)
Age groups (%)	
18–34	30 (0.7)
35–44	51 (1.2)
45–54	129 (3.0)
55–64	350 (8.1)
65–74	726 (16.9)
75–84	1516 (35.3)
>85	1498 (34.8)
Female (%)	2225 (51.7)
Length of stay, Days (SD)	10.78 (7.12)
Age of death (SD)	78.84 (12.35)
Hypertension (%)	3293 (76.6)
Solid neoplasm (%)	2783 (64.7)
Ischemic Heart Disease (%)	2034 (47.3)
Diabetes (%)	1968 (45.8)
Chronic Kidney Disease (%)	1822 (42.4)
Acute Heart Failure (%)	1549 (36.0)
Atrial Fibrillation (%)	1447 (33.7)
Congestive Heart Failure (%)	1356 (31.5)
Chronic Obstructive Pulmonary Disease (%)	913 (21.2)
Cerebrovascular Accident (%)	508 (11.8)
Asthma (%)	419 (9.7)
Hematologic Neoplasm (%)	323 (7.5)
Cirrhosis (%)	150 (3.5)

**Table 2:** Association Between Co-Morbidities and In-Hospital Mortality by Age Group (18–64): Logistic Regression Analysis.

(**a**)												
	**Age Group 18–34**			**Age Group 35–44**			**Age Group 45–54**			**Age Group 55–64**		
**Characteristic**	**OR ^1^**	**95% CI ^2^**	***p*** **-Value**	**OR ^1^**	**95% CI ^2^**	***p*** **-Value**	**OR ^1^**	**95% CI ^2^**	***p*** **-Value**	**OR ^1^**	**95% CI ^2^**	***p*** **-Value**
Male	0.86	0.33, 2.10	0.7	1.37	0.71, 2.69	0.3	0.86	0.55, 1.33	0.5	1.08	0.82, 1.42	0.6
DM ^1^	1.74	0.33, 7.22	0.5	1.18	0.43, 2.99	0.7	0.77	0.47, 1.23	0.3	0.89	0.67, 1.20	0.4
IHD ^2^	0.00		>0.9	0.41	0.06, 1.86	0.3	1.38	0.73, 2.57	0.3	1.05	0.75, 1.45	0.8
CVA ^3^	16.0	0.81, 437	0.057				1.59	0.60, 3.93	0.3	2.10	1.34, 3.27	0.001
CHF ^4^	4.66	0.25, 168	0.3				1.54	0.42, 6.59	0.5	1.28	0.68, 2.44	0.4
Hemato neoplasm	10.0	2.13, 48.7	0.003	1.50	0.42, 4.60	0.5	2.55	1.24, 5.16	0.010	1.25	0.80, 1.94	0.3
Solid neoplasm	3.18	1.30, 8.20	0.013	1.52	0.77, 3.00	0.2	1.51	0.98, 2.35	0.062	1.44	1.08, 1.92	0.013
Hypertension	1.51	0.27, 6.74	0.6	1.16	0.41, 3.04	0.8	0.94	0.57, 1.56	0.8	0.82	0.60, 1.12	0.2
COPD ^5^	1.13	0.04, 18.8	>0.9	0.88	0.17, 3.39	0.9	2.58	1.45, 4.61	0.001	1.40	0.99, 1.98	0.058
Asthma	0.75	0.11, 3.31	0.7	1.50	0.49, 4.12	0.4	0.47	0.20, 1.01	0.063	0.85	0.52, 1.36	0.5
Acute HF ^6^	3.95	0.15, 44.9	0.3	2.03	0.61, 6.32	0.2	1.0	0.25, 3.24	>0.9	1.22	0.67, 2.17	0.5
AF ^7^	0.77	0.03, 9.89	0.9	0.46	0.02, 2.98	0.5	1.60	0.77, 3.26	0.2	1.22	0.83, 1.76	0.3
CKD ^8^	0.60	0.13, 2.18	0.5	0.31	0.09, 0.94	0.053	0.63	0.37, 1.04	0.078	1.50	1.11, 2.01	0.007
Cirrhosis	0.32	0.01, 2.86	0.4	0.70	0.04, 4.44	0.7	4.09	1.79, 9.41	<0.001	2.44	1.40, 4.26	0.002
(**b**)												
	**Age Group 65–74**				**Age Group 75–84**				**Age Group > 85**			
**Characteristic**	**OR ** ^1^	**95% CI ** ^2^		***p*** **-Value**	**OR ** ^1^	**95% CI ** ^2^		***p*** **-Value**	**OR ** ^1^	**95% CI ** ^2^		***p*** **-Value**
Male	0.90	0.74, 1.10		0.3	0.91	0.79, 1.06		0.2	0.85	0.72, 1.00		0.045
DM ^1^	1.17	0.95, 1.43		0.14	0.95	0.82, 1.10		0.5	1.00	0.85, 1.18		>0.9
IHD ^2^	0.98	0.78, 1.22		0.8	0.98	0.84, 1.15		0.8	1.15	0.98, 1.36		0.092
CVA ^3^	1.30	0.96, 1.76		0.090	1.19	0.95, 1.49		0.13	1.36	1.04, 1.77		0.025
CHF ^4^	1.53	0.96, 2.48		0.079	1.57	1.10, 2.26		0.014	0.89	0.59, 1.33		0.6
Hemato neoplasm	1.84	1.30, 2.60		<0.001	1.33	1.00, 1.76		0.048	1.28	0.86, 1.93		0.2
Solid neoplasm	0.93	0.75, 1.14		0.5	1.01	0.87, 1.18		0.9	0.88	0.74, 1.04		0.12
Hypertension	0.74	0.58, 0.94		0.014	1.03	0.85, 1.25		0.8	1.05	0.86, 1.28		0.6
COPD ^5^	1.35	1.05, 1.74		0.019	1.06	0.88, 1.29		0.5	1.04	0.84, 1.30		0.7
Asthma	0.91	0.64, 1.29		0.6	1.07	0.83, 1.37		0.6	1.05	0.78, 1.43		0.7
Acute HF ^6^	0.93	0.58, 1.46		0.7	0.93	0.66, 1.32		0.7	1.44	0.97, 2.15		0.074
AF ^7^	1.21	0.96, 1.53		0.10	1.41	1.20, 1.66		<0.001	1.04	0.88, 1.23		0.7
CKD ^8^	1.19	0.97, 1.47		0.10	1.02	0.87, 1.19		0.8	1.03	0.87, 1.21		0.7
Cirrhosis	1.89	1.16, 3.08		0.010	1.57	1.00, 2.51		0.052	1.48	0.64, 3.67		0.4

^1^ OR = Odds Ratio, ^2^ CI = Confidence Interval; Reference category for all comorbidities: absence of the condition; for gender: male; ^1^ DM= Diabetes melitus; ^2^ IHD = Ischemic Heart Disease; ^3^ CVA = Cerebrovascular Accident; ^4^ CHF = Congestive Heart Failure; ^5^ COPD = Chronic Obstructive Pulmonary Disease; ^6^ HF = Heart Failure; ^7^ AF = Atrial Fibrillation; ^8^ CKD = Chronic Kidney Disease. Significant parameters are underlined.

## Data Availability

The data that support the findings of this study are not publicly available due to patient privacy and institutional restrictions.

## Supplementary Materials

The following supporting information can be downloaded at: https://www.mdpi.com/article/10.3390/jcm14217637/s1, Figure S1: AIC values for spline and polynomial models by age group; Table S1: ICD-9 Codes Used to Identify Sepsis Cases.
