# Supplementary material for: The Impact of Age on In-Hospital Mortality in Patients with Sepsis: Findings from a Nationwide Study
Source: J Clin Med. 2025 Oct 28;14(21):7637. doi: 10.3390/jcm14217637 (PMC12610147; doi:10.3390/jcm14217637)
Supplement: Supplementary file 1 [file jcm-14-07637-s001.zip › jcm-3864233-supplementary.pdf]

Supplementary Figure S1. AIC values for spline and polynomial models by age group

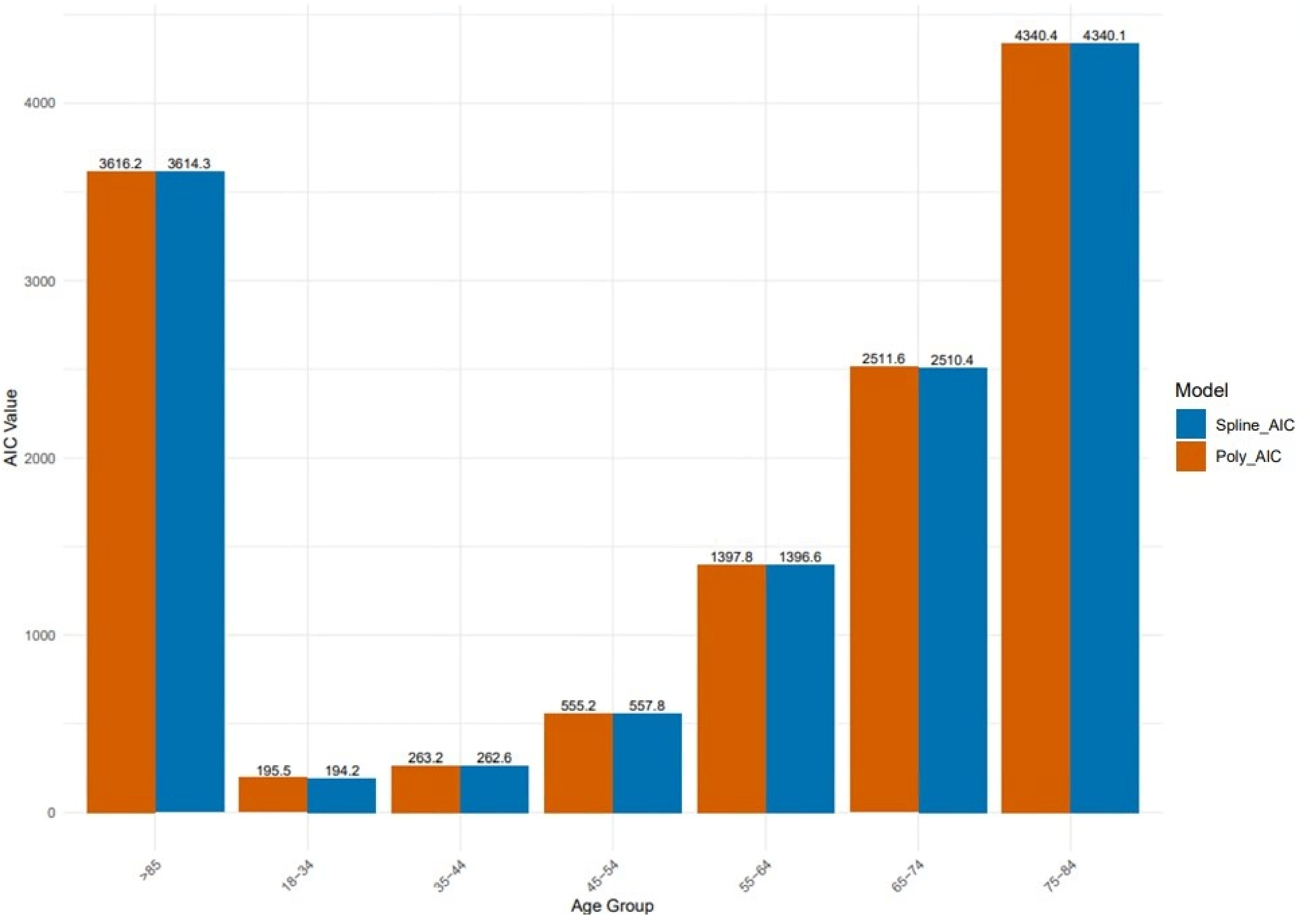

Supplemental Table S1. ICD-9 Codes Used to Identify Sepsis Cases

ICD-9 Code	Condition / Description
003.1	Salmonella septicemia
020.2	Septicemic plague
022.3	Anthrax septicemia
036.2	Meningococcal septicemia
036.3	Waterhouse–Friderichsen syndrome
038.0	Streptococcal septicemia
038.1	Staphylococcal septicemia
038.2	Pneumococcal septicemia
038.3	Septicemia due to anaerobes

ICD-9 Code	Condition / Description
038.4	Septicemia due to Gram-negative organisms
038.8	Other specified septicemias
038.9	Unspecified septicemia
054.5	Herpetic septicemia
098.89	Other specified gonococcal infections (with sepsis)
112.5	Disseminated candidiasis
995.91	Sepsis
995.92	Severe sepsis
785.52	Septic shock
